# Acute Presentation of Rapunzel Syndrome and a Review of Bezoars

**DOI:** 10.7759/cureus.20785

**Published:** 2021-12-28

**Authors:** Xinlin Chin, Jessica Y Ng

**Affiliations:** 1 Surgery, Mackay Base Hospital, West Mackay, AUS; 2 School of Medicine, Griffith University, Birtinya, AUS; 3 School of Medicine, Griffith University, Southport, AUS

**Keywords:** bezoar, trichotillomania, trichophagia, rapunzel syndrome, trichobezoar, gastric bezoar

## Abstract

Bezoars have different compositions and can be subdivided into trichobezoar, phytobezoar, pharmacobezoar, lactobezoar and food bolus. The reported incidence of bezoar is 0.4% with phytobezoar being the commonest. Rapunzel syndrome is an extremely rare complication when trichobezoar crosses the pylorus to enter the duodenum, ileum and colon. We present the case of a 29-year-old female with a one-week history of abdominal pain, anorexia, nausea, vomiting, constipation, lethargy and a one-year history of increasing abdominal mass. Physical examination revealed a 20 cm palpable mass extending from the left upper quadrant to the umbilicus. Laboratory investigations demonstrated iron deficiency anemia and CT showed two well-defined foci within the gastric lumen consistent with trichobezoars. She was managed conservatively during her hospital stay and discharged home with a plan for elective laparotomy. We present this case to discuss the management of trichobezoars and to highlight the importance of early recognition of recurrence to avoid severe complications.

## Introduction

Bezoar, derived from the Arabic word ‘Bazahr’, refers to an agglutination of indigestible content in the gastrointestinal tract [1-3]. Bezoars have different compositions and can be subdivided into trichobezoar (hair ball), phytobezoar (plant), pharmacobezoar (medicinal preparation), lactobezoar (milk curds) and food bolus [1, 4-6]. The reported incidence of bezoar is 0.4% with phytobezoar being the commonest [6]. Trichobezoars are usually seen in females under the age of 30 and may achieve a mortality rate of 30% if left untreated [2, 7]. Trichobezoar is usually secondary to trichotillomania and trichophagia with scalp, eyebrows and eyelashes being the commonest sites [3, 5]. Atypical dietary habits, impaired mastication, diabetic gastroparesis, achlorhydria and gastric dysmotility secondary to gastrectomies may also contribute to the formation of trichobezoars [1].

## Case presentation

A 29-year-old female presented to the emergency department with a one-week history of worsening left upper quadrant (LUQ) pain, anorexia, nausea, vomiting, constipation and lethargy. She had a four-year history of ongoing abdominal pain and a one-year history of an increasing abdominal mass. Her past medical history was significant for trichotillomania, trichophagia and anxiety which started four years prior in the context of relationship stressors. She reported no surgical history. Vital signs were stable. No obvious alopecia or halitosis were noted. Physical examination revealed a soft abdomen with LUQ tenderness and a firm palpable mass (approximately 20 cm) extending from the LUQ to the umbilicus.

Laboratory investigations showed a reduced haemoglobin level of 63 g/L, mean corpuscular volume of 78 fL and an elevated white cell count of 17.7 × 109/L. Urea, electrolytes and liver function tests were normal. C-reactive protein was elevated at 104 mg/L. Iron studies demonstrated marked iron deficiency with an iron level of less than 2 umol/L, ferritin 11 ug/L and transferrin saturation of <4%. Vitamin B12 and folate levels were within the normal range but a low albumin level of 26 g/L was noted.

**Figure 1:**
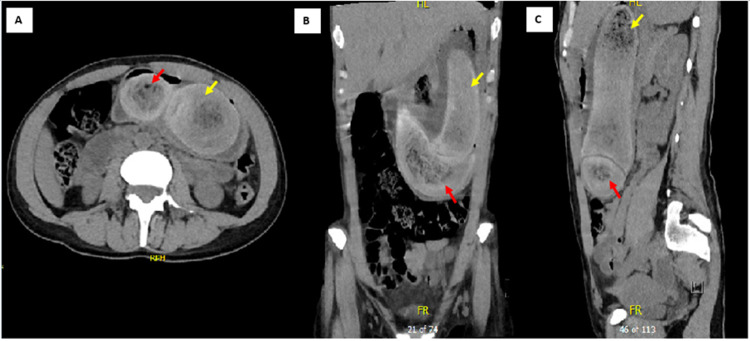
Axial (A), coronal (B) and sagittal (C) computed tomography imaging of the abdomen and pelvis showed two well-defined foci in the distended stomach. The larger focus (yellow arrow) is seen within the gastric lumen while the second focus (red arrow) extends distally into the pyloric antrum

Computed tomography imaging of the abdomen and pelvis (CTAP) showed two well-defined foci of heterogeneous density but predominantly hyperdense foreign material within the gastric lumen consistent with trichobezoars. The larger focus (18 x 6 x 5.6 cm) is seen in the distended gastric body while the second focus (12 x 4.7 x 4.5 cm) extends from the distal gastric body into the pyloric antrum. There were no signs of gastric outflow obstruction and perforation (Figure 1). Endoscopy was not performed given the characteristic radiological findings of trichobezoar on CTAP and history of trichophagia. Endoscopy was not attempted due to the sizes of trichobezoars.

**Figure 2:**
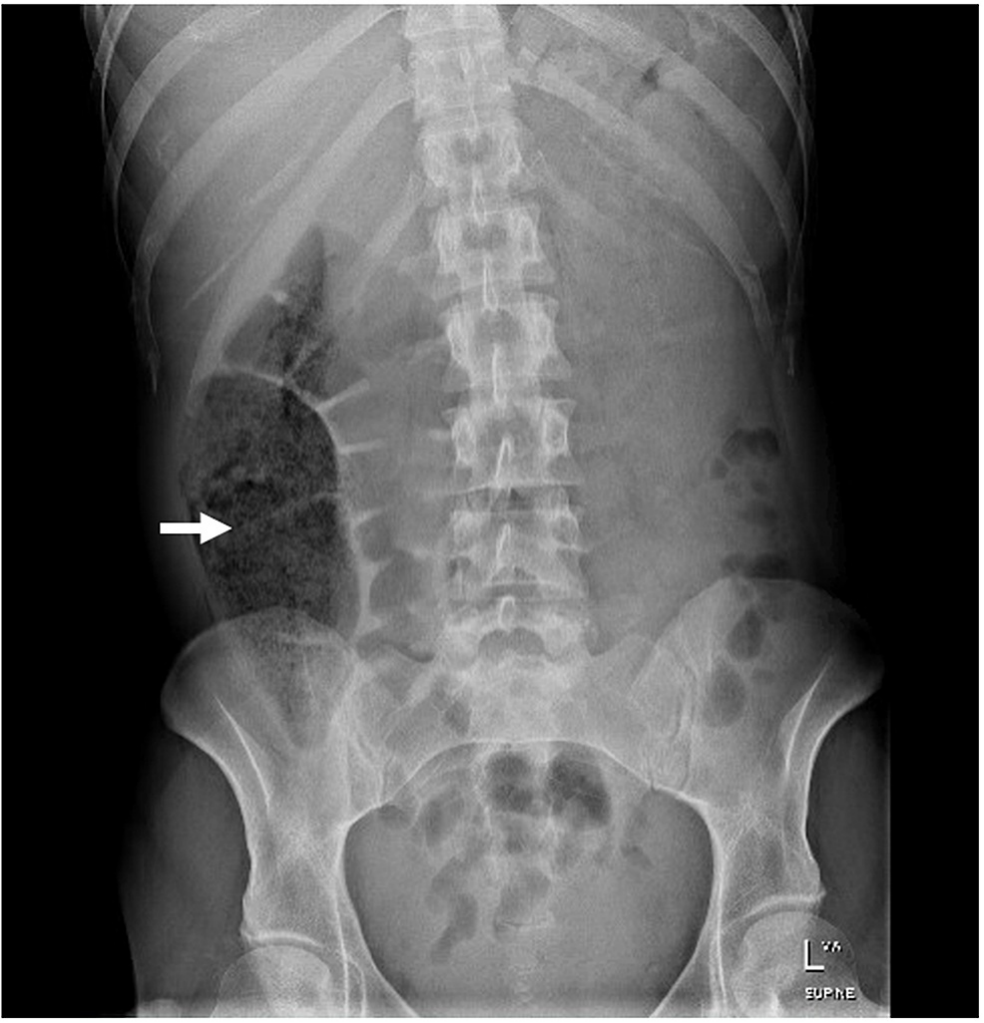
Abdominal X-ray showed progression of the trichobezoar into the caecum and ascending colon

The patient was fasted and commenced on intravenous fluids and intravenous pantoprazole 40 mg twice a day. Two units of iron and three units of packed red blood cells were transfused in view of iron deficiency anemia secondary to chronic malnutrition. Multivitamins with minerals (one tablet daily) and oral thiamine (100 mg daily) were prescribed for malnutrition. She was managed conservatively with Coca-Cola consumption twice a day for at least four days (approximately 2400 ml in total) and was commenced on a free fluid diet. Regular Macrogol-3350 and Coloxyl-Senna were used to aid with bowel motions while her abdominal pain was well managed with intravenous hyoscine butylbromide. Abdominal X-ray taken four days later demonstrated progression of the trichobezoars into the caecum and ascending colon (Figure 2). She returned to full diet and gastrointestinal function after five days of hospital stay. Dietetics input for diet modification and nutritional support along with psychiatric consultation for trichotillomania management were obtained throughout her hospital admission.

She was discharged home with oral ferrous fumarate tablets and a plan for elective laparotomy when she is medically and mentally optimized. She was scheduled for outpatient follow-up with the general surgeons, dietician and psychiatrist for ongoing review.

## Discussion

Trichobezoars resist peristalsis and retain in the gastric rugae due to their smooth surface and poorly digested keratinaceous substance [8]. Gastric acid denatures hair proteins and oxidizes the hair, giving trichobezoars a dark colour [9]. The hair which escaped propulsion is matted into a ball along with accumulated food particles, causing halitosis due to bacterial colonization and undigested fat fermentation [9].

The clinical presentation of trichobezoars is associated with its size and presence of complications [3]. 33 to 37% of the patients present with common symptoms like abdominal pain, nausea and vomiting while some patients may remain asymptomatic for years [3, 5, 7]. 85% of the patients demonstrate a well-defined, firm and mobile mass in the epigastrium while 20% may be peritonitic upon physical examination [3, 5]. Severe halitosis and patchy alopecia are helpful clues for the diagnosis of trichobezoar [7].

Rapunzel Syndrome is an extremely rare complication where trichobezoars cross the pylorus to enter the duodenum, ileum and colon [1, 5]. Fewer than a hundred cases were reported since its first description in 1968 [5]. The extension of trichobezoars into the cecum and ascending colon as shown in our patient’s abdominal X-ray supports the diagnosis of Rapunzel Syndrome. Other complications include bowel obstruction, perforation, intussusception, malnutrition, haematemesis and obstructive jaundice [1-3, 5].

Conservative management of trichobezoars includes hydration, Coca-Cola dissolution, meat tenderizers and prokinetic agents [1, 10]. Coca-Cola with a pH of 2.6 dissolves fibre, shrinks and softens the bezoars [6]. While sodium bicarbonate and carbon dioxide bubbles enhance Coca-Cola’s dissolving mechanism, the carbon dioxide bubbles may increase gastric pressure causing post-pyloric extension of the shrunken bezoar leading to bowel obstruction [11].

Exploratory laparotomy is the standard treatment for trichobezoar and remains the only valid treatment for Rapunzel Syndrome as it has a shorter operative duration, allows feasible examination of the gastrointestinal tract and enables definitive management when complications arise [3, 7]. The success rate is near 100% with a complication rate of 12-14% owing to occasional intra-abdominal spillage and wound infection [7]. Laparoscopic removal has a success rate of 75% with an increased risk of peritoneal contamination as the bezoar is transferred within the peritoneal cavity [7, 8]. Although endoscopy provides a reliable diagnosis of intragastric trichobezoar, it has a low therapeutic success rate of 5% and is restricted to trichobezoars smaller than 55 g or less than 2 to 3 cm in diameter [3, 5, 7, 10]. 

As patients with trichobezoars may have vague presentations, recurrence features such as anemia, weight loss, abdominal mass, malnutrition and parental concern should prompt early consideration of endoscopic evaluation [3, 4]. There is a lack of guidelines on post-operative follow-up appointments for patients to reduce the risk of recurrence of bezoars but routine ultrasound or endoscopy at the 6th, 12th and 24th months in the post-operative period have been suggested for surveillance [3]. A multidisciplinary approach with dietetics, psychiatric involvement for trichotillomania management along with a good support network is important to reinforce treatment and prevent recurrence [1, 3].

## Conclusions

Trichobezoars and Rapunzel Syndrome are rare in clinical practice and require urgent intervention to avoid severe complications. Coca-Cola or meat tenderizers may be considered as an option for conservative management of trichobezoars, however, exploratory laparotomy is the standard management and remains the only valid treatment for Rapunzel Syndrome. As patients with trichobezoar may have a vague presentation and are prone to recurrence, a multidisciplinary approach with dietetics, long-term psychiatric follow-up for trichotillomania management, a good support network and close monitoring are important for early detection and recurrence prevention. Further research on the follow-up appointments required for post-operative patients may be considered to reduce recurrence and facilitate early detection.

## References

[1] Amjad W, Upadhya G, Hurairah A, Iqbal S (2017). Endoscopic shaving of hair in a gastric bypass patient with a large bezoar. BMJ Case Rep.

[2] Kumar N, Huda F, Gupta R, Payal YS, Kumar U, Mallik D (2019). Rapunzel syndrome in adult with mysterious presentation: a rare case report with literature review. Trop Doct.

[3] Lyons R, Ismaili G, Devine M, Malik H (2020). Rapunzel syndrome causing partial gastric outlet obstruction requiring emergency laparotomy. BMJ Case Rep.

[4] Gorter RR, Kneepkens CM, Mattens EC, Aronson DC, Heij HA (2010). Management of trichobezoar: case report and literature review. Pediatr Surg Int.

[5] Hamid M, Chaoui Y, Mountasser M (2017). Giant gastric trichobezoar in a young female with Rapunzel syndrome: case report. Pan Afr Med J.

[6] Ladas SD, Kamberoglou D, Karamanolis G, Vlachogiannakos J, Zouboulis-Vafiadis I (2013). Systematic review: Coca-Cola can effectively dissolve gastric phytobezoars as a first-line treatment. Aliment Pharmacol Ther.

[7] Darwish AA, Abdelgawad AE, Platt E, Garrett-Cox R (2019). Formation of a temporary gastrostomy to aid delivery of gastric trichobezoar and decrease incidence of wound infection. BMJ Case Rep.

[8] Tudor EC, Clark MC (2013). Laparoscopic-assisted removal of gastric trichobezoar; a novel technique to reduce operative complications and time. J Pediatr Surg.

[9] Nour I, Abd Alatef M, Megahed A, Yahia S, Wahba Y, Shabaan AE (2019). Rapunzel syndrome (gastric trichobezoar), a rare presentation with generalised oedema: case report and review of the literature. Paediatr Int Child Health.

[10] Zarling EJ, Moeller DD (1981). Bezoar therapy. Complication using Adolph's Meat Tenderizer and alternatives from literature review. Arch Intern Med.

[11] Lu L, Zhang XF (2016). Gastric outlet obstruction--an unexpected complication during Coca-Cola therapy for a gastric bezoar: a case report and literature review. Intern Med.

